# A novel strategy for clustering major depression individuals using whole-genome sequencing variant data

**DOI:** 10.1038/srep44389

**Published:** 2017-03-13

**Authors:** Chenglong Yu, Bernhard T. Baune, Julio Licinio, Ma-Li Wong

**Affiliations:** 1 Mind and Brain Theme, South Australian Health and Medical Research Institute, North Terrace, Adelaide, SA 5000, Australia; 2 School of Medicine, Flinders University, Bedford Park, SA 5042, Australia; 3 Discipline of Psychiatry, School of Medicine, University of Adelaide, Adelaide, SA 5005, Australia

## Abstract

Major depressive disorder (MDD) is highly prevalent, resulting in an exceedingly high disease burden. The identification of generic risk factors could lead to advance prevention and therapeutics. Current approaches examine genotyping data to identify specific variations between cases and controls. Compared to genotyping, whole-genome sequencing (WGS) allows for the detection of private mutations. In this proof-of-concept study, we establish a conceptually novel computational approach that clusters subjects based on the entirety of their WGS. Those clusters predicted MDD diagnosis. This strategy yielded encouraging results, showing that depressed Mexican-American participants were grouped closer; in contrast ethnically-matched controls grouped away from MDD patients. This implies that within the same ancestry, the WGS data of an individual can be used to check whether this individual is within or closer to MDD subjects or to controls. We propose a novel strategy to apply WGS data to clinical medicine by facilitating diagnosis through genetic clustering. Further studies utilising our method should examine larger WGS datasets on other ethnical groups.

With the development of new and cheaper whole genome sequencing (WGS) technology, patient care may move towards precision medicine. Ever since the first human genome was fully sequenced, scientists have been searching for approaches to provide personalized care^1^. WGS allows us to identify single nucleotide variants (SNVs), which are private genetic variants, and determine all the genetic variants within each person. Single nucleotide polymorphism (SNP) genotyping is currently the gold-standard technique for genome-wide association studies (GWAS), as WGS costs remain relatively high; however, as WGS costs are projected to drop further, researchers may have the opportunity to examine the significance of SNVs, which involve more individual characteristics.

Major depressive disorder (MDD) is a chronic condition with great medical, social, and economic impacts. MDD is a main contributor to global disease burden and produces significant morbidity and mortality^2^,^3^,^4^,^5^,^6^. Despite recent advances^7^,^8^,^9^, little is known about its underlying fundamental biology. The existing psychiatric genetic studies have not found common consistently replicated gene variants of large effect in the pathogenesis of MDD^10^,^11^,^12^, and thus much work still needs to be done to fully elucidate the genetic factors that confer susceptibility to this condition. For our current research, we tested whether the combined effect of all SNVs at the whole-genome sequence level could confer genetic liability to the MDD risk.

In this study, we focus on a sample of Los Angeles Mexican-American participants who had three or more grandparents born in Mexico. MDD participants were diagnosed using the Structured Clinical Interview (SCID) for Diagnostic and Statistical Manual of Mental Disorders (DSM), and the DSM-IV diagnostic criteria for current, unipolar major depressive episode with a HAM-D21 (21-Item Hamilton Depression Rating Scale) score of 18 or greater with item number 1 (depressed mood) rated 2 or greater; they participated in a pharmacogenetic study of antidepressant treatment. Controls were in general good health but were not screened for medical or psychiatric illnesses; they were age- and gender- matched Mexican-American individuals recruited from the same community in Los Angeles^13^,^14^,^15^,^16^. Here, we establish a new computational approach to cluster subjects based on all of their WGS variants. We believe that clustering of patients based on their SNV profiles may provide valuable clues for prognostics, diagnostics, and therapeutics, as it takes into account all of their genetic data. The idea for this approach arose from distance-based phylogenetic analysis of DNA/protein sequences proposed by us earlier^17^,^18^,^19^,^20^,^21^. In our proposed methodology we used a well-defined metric in mathematics, the Jaccard distance, to measure the similarity/dissimilarity between subjects using all the SNV information from each individual and from that we construct cluster trees based on the Jaccard distance matrices. Clustering relationships in the trees showed that Mexican-American MDD patients grouped together, and were clustered far from ethnically matched healthy controls. This discovery may be translated to clinical practice since we may be able to predict the MDD status of a given Mexican-American subject based on his/her WGS data.

## Materials and Methods

### The Mexican-American Sample

In our recent work^16^, we have investigated the whole-exome genotyping data of a Los Angeles Mexican-American cohort aged 19–65 years of 203 MDD patients and 196 healthy controls. Participants provided written informed consent, and detailed demographic, epidemiological, and clinical descriptions were previously described^13^,^14^,^15^. The study was registered in ClinicalTrials.gov (NCT00265291), and approved by the Institutional Review Boards of the University of California Los Angeles and University of Miami, USA, and by the Human Research Ethics Committees of the Australian National University and Bellbery Ltd, Australia^13^,^14^,^15^. In this study, we obtained complete WGS data for a group of 15 participants selected from the cohort, 10 MDD patients and 5 controls. In Table 1, we present the gender (all are female) and age information of the 15 Mexican-American subjects. We have confirmed that in the cohort there was no family or population structure among all those individuals^16^ and no any blood relationship among the 15 selected participants.

### The European-Ancestry Sample

For comparison as an outgroup sample, we also include WGS data from a group of 10 Australians of European-Ancestry. Those 10 participants gave written informed consent and were recruited under the Cognitive function and mood disorders study (conducted by the Discipline of Psychiatry, University of Adelaide, South Australia, Australia). This sample was studied under approved Human Research Ethics Committees protocols at the University of Adelaide and Flinders University, South Australia, Australia. In Table 1, we present the gender and age information of these 10 subjects.

We confirm all methods and experiments in this study were performed in accordance with relevant guidelines and regulations.

### Whole-Genome Sequencing (WGS) and Analysis

Samples from fifteen Mexican-American participants (10 MDD patients and 5 controls) were whole-genome sequenced using Illumina HiSeq 2000 (BGI-Shenzhen, Shenzhen, Guangdong, China) and samples from ten European-Ancestry Australian participants (5 MDD patients and 5 controls) were whole-genome sequenced using HiSeq X (Garvan Institute, Sydney, New South Wales, Australia). After obtaining paired-end sequencing reads of those 25 participants, we did the variant calling analysis using the following pipeline. The reads of each participant were aligned to the human reference genome (hg19, Genome Reference Consortium GRCh37) using Burrows-Wheeler Aligner (BWA)^22^ to get SAM (sequence alignment/map) format files. SAM files were converted to the BAM (binary version of a SAM file) format files using SAMtools^23^. BAM files were then merged into one BAM file, and the mpileup command in SAMtools was used to calculate the likelihood of data given each possible genotype, and store the likelihoods in a binary file. The output was supplied to SAMtools/BCFtools^24^ which created the SNV/INDEL (small insertions and deletions) calling to generate VCF (variant call format) files. Then, ANNOVAR^25^ was used to annotate SNV/INDEL information and their classifications. For WGS and analysis details, please see Supplementary Information. Only the SNV information was used in the following methodology.

### Clustering Subjects on SNV Sets

**Figure 1:**
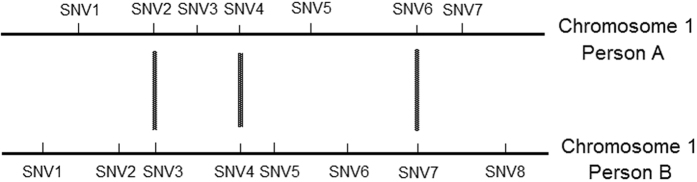
A hypothetical illustration of the distribution of SNVs on chromosome (chr) 1 of two individuals. SNV2, SNV4 and SNV6 in person A and SNV3, SNV4 and SNV7 in person B occupy the same respective positions in chr 1.

To take in consideration all the SNV information of those subjects, it was important to define a distance between two subjects when running the cluster analysis. We clustered the subjects at the chromosome level; consequently, we defined a distance between two people based on SNV information obtained from a given chromosome, e.g., chromosome 1. First, we considered the SNVs distribution on that chromosome. In Fig. 1, we give a hypothetical SNVs distribution on chromosome 1 for two individuals. In a real case scenario, in a given chromosome two individuals may have many same position SNVs, e.g., SNV6 in person A and SNV7 in person B in Fig. 1. Our hypothesis was that if two individuals shared more same position SNVs, then those two individuals would have more similar phenotypes, such as traits or diseases. Therefore, a proper distance (dis-similarity) between two SNV sets could be employed.

Let *S*_1_ and *S*_2_ be SNV sets in a given chromosome from subject 1 and subject 2. We use |*S*| to denote the cardinality of set *S*. The Jaccard metric^26^, a statistics tool for measuring the similarity and diversity of sample sets, is introduced here. The Jaccard metric between *S*_1_ and *S*_2_ is defined as


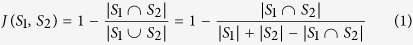


If *S*_1_ and *S*_2_ are exactly the same, then 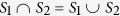, *J(S*_1_, *S*_2_) = 0. If *S*_1_ and *S*_2_ have no common elements, then 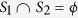, *J(S*_1_, *S*_2_) = 1. Unlike many distances used in comparative genomics (e.g., some distances based on alignment methods), the Jaccard metric satisfies the triangle inequality, i.e., 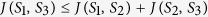, which guarantees that the Jaccard distance is a well-defined metric in mathematics^27^. Considering Fig. 1 as an example, *S*_*A*_ = 7, *S*_*B*_ = 8, 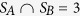, so


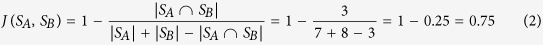


In this proof-of-principle study we use the Jaccard metric to calculate the distance matrices of those 25 participants in each chromosome. Then cluster trees based on each chromosome can be reconstructed. Clustering relationships shown in the trees may reveal significant medical information that may be translated into clinical practice.

## Results

### Whole-Genome Sequencing (WGS) Data Analysis

Table 1 provides the results of WGS variation in 25 human subjects and shows that Mexican-American individuals have significantly more SNVs when compared to Australian individuals of European-Ancestry. For total SNVs, Australian’s mean value is 3901078 (*n = *10), Mexican-American’s mean value is 7729021.3 (*n* = 15), the *t* test *p*-value for the two groups is 2.09e-15. This is consistent with data from the Human Haplotype Matching Project (HapMap). We contributed the Mexican-American sample to HapMap, from the same community as subjects in this study. That study showed that Mexican-Americans have more polymorphic SNPs in Mexican-Americans than in northern Europeans^28^. Mexican-Americans from that Los Angeles community have median ancestry proportions that are 45% Indigenous American, 49% European and 5% African^29^. According to results from the International HapMap 3 Consortium and the 1000 Genomes Project Consortium, it would be expected that individuals with African ancestry, such as Mexican-Americans, have increased number of variants, and, moreover, the Spanish population have excess of rare variants^28^,^30^.

**Figure 2:**
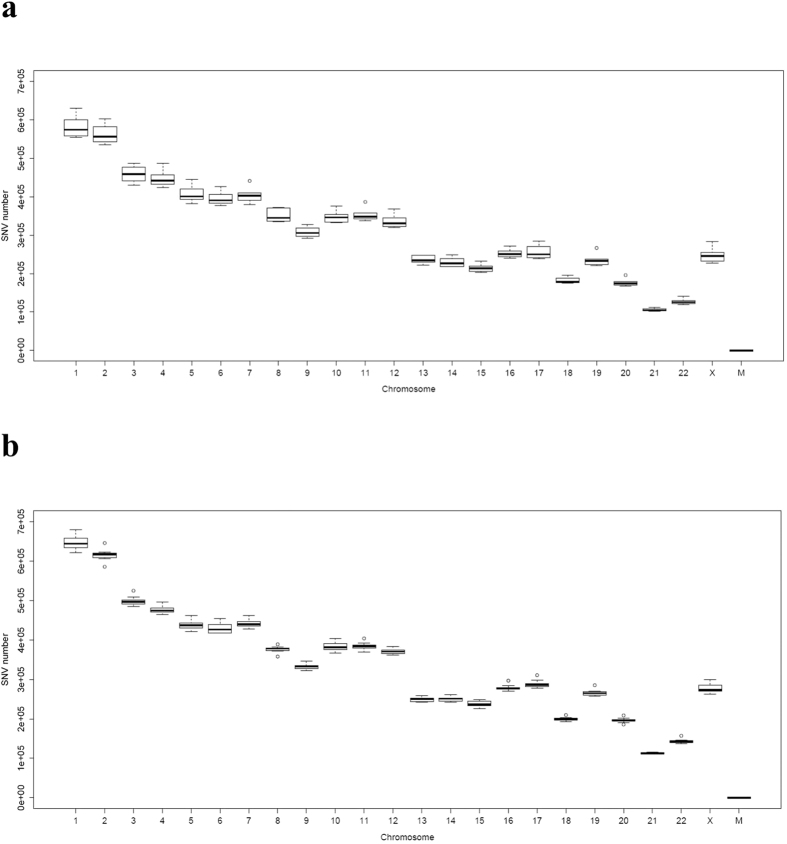
Descriptive statistical distributions of SNVs on all the chromosomes. (**a**) The Mexican-American control group. (**b**) The Mexican-American MDD group.

For the Mexican-American sample, both depression and control subjects have approximately 7,000,000 to 8,000,000 SNVs; 5,100,000 to 5,200,000 INDELs, and 3,900,000 to 4,000,000 SNVs in dbSNP (the SNP database). We calculated the SNV distributions on each chromosome for the Mexican-American and Australian samples. In Fig. 2a, we used boxplot to show the descriptive statistical distributions of SNVs in each chromosome for the Mexican-American control group. Descriptive statistical distributions of SNVs of each chromosome for the Mexican-American depression group are provided in Fig. 2b. Since only female Mexican-American samples were used for this study, we include chromosome X in the results. We found that the depression and control groups have basically the same SNV distributions for all chromosomes. Table S1 provides detailed information of SNV distributions for all the chromosomes in the 25 subjects.

### Clustering Subjects using Cluster Trees

**Figure 3:**
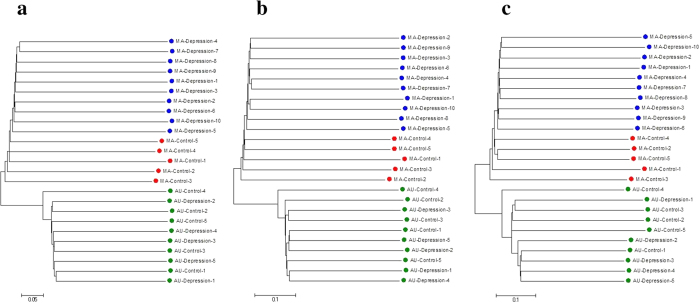
Cluster tree of 25 subjects for different chromosomes. (**a**) On chromosome 1. (**b**) On chromosome 22. (**c**) On chromosome X.

Following the proposed method, we use the Jaccard metric and SNV sets to obtain the distance matrices between those 25 participants for each chromosome. Jaccard distance calculation was done using R programming language. We used the popular neighbor-joining method^31^ on the distance matrices to construct cluster trees, which were drawn using software MEGA 6^32^. Figure 3a shows the cluster tree for 25 subjects in chromosome 1. We found that all the 10 Mexican-American MDD patients grouped together in a cluster, and 5 Mexican-American controls were separated from that group. The Australian individuals of European-Ancestry, as a different population, assembled as an obvious outgroup from the Mexican-American subjects. This fact is also consistent with the genetic distance between different populations^33^. We constructed cluster trees for all chromosomes. Except for the mitochondrial genome, all cluster trees clustered the Mexican-American MDD patients as group distinct from the controls. Although the Australian subjects stably stand as an outgroup, within that group the MDD and control individuals could not be well distinguished as in the Mexican-American group. Figure 3b and c show the cluster trees in chromosome 22 and chromosome X, respectively. In Figure S1, we provided the cluster trees for all the other 20 chromosomes and mitochondrial genome.

WGS data analysis and Jaccard distance calculations were performed using high-performance computers in eResearch South Australia (https://www.ersa.edu.au/).

## Discussion

The results obtained by our new approach support the assumption that two individuals who share more of the same position SNVs would have more similar phenotypes, such as traits or diseases. Clustering relationships in the trees show that the Mexican-American MDD patients group together, and ethnically matched controls grouped away and separately. The fact that Australian subjects fail to be clustered into case and control groups may imply that this computational method may be restricted to specific populations, with a higher degree of genetic diversity, such as Mexican-Americans. It should be noted that the choice of Jaccard metric was not random. When measuring similarity between two SNV sets, the intersection of two sets denotes the shared same position SNVs of two people, and the union of two sets is used to normalize the similarity to a value between 0 and 1. All the SNV information for two sets is fully utilized in this metric. Furthermore, the Jaccard metric is a rigorous mathematical distance. Our results showed that it is appropriate to cluster Mexican-American MDD subjects in this study. Among distance-based tree construction methods, the neighbor-joining technique does not assume a constant rate of evolution, as opposed to the molecular clock hypothesis. Due to its low computational complexity it can be performed quickly and is widely used to generate cluster trees of individuals^34^,^35^.

We have confirmed that there were no blood relatives between those Mexican-American subjects, thus the clustering relationships in the trees were not due to genetic relatedness. For the Mexican-American sample, all the subjects were female, and the MDD case group had an average age of 38.8 years with standard deviation 8.15 and the control group had an average age of 39.6 years with standard deviation 7.36. The two groups have basically the same age distribution. Thus the clustering results were not associated with gender and age. For our approach, confounding phenotypes with complex genetic architecture may be reflected in the measured distance and this could alter the observed clustering. Therefore, before performing our method, it is necessary to control confounding factors such as ethnicity, MDD diagnostic and control selection criteria, genetic relatedness, gender and age.

Our aim in this paper was not to confirm or refute previous genetic research of depression such as candidate gene studies or GWAS^36^ but rather to bring a novel direction using comparative genomic analysis at the whole-genome sequence level. In our methodology, the combined effect of all SNVs in the complete genome, including all genomic regions such as coding and non-coding, was considered as a genetic factor to the depression risk. Our computational approach allowed us to perform a global comparison of whole-genome information in the subjects, which no other existing method can achieve. Once a Jaccard distance matrix has been constructed, the results in the clustering tree can be displayed and viewed graphically; this is user-friendly and allows even non-expert to understand the relationships among the subjects. Furthermore, most existing genome-wide analysis methods involve many statistical models. The different choices of these models can lead to inconsistent results. Our method does not involve any statistical model and it depends only on the genetic distance between two individuals by considering their whole-genome SNV information. Therefore, our approach is stable and produces a unique analysis result.

High quality full genome sequencing costs are currently still a concern that limits obtaining larger datasets; another limitation is the high level of computational resources needed for sequencing data analysis. Future studies utilising our method should examine additional replication data on other ethnical groups.

We have developed a novel methodology to cluster subjects based on their WGS data. To the best of our knowledge, this is the first time that SNV and cluster analysis are used to study major depressive disorder. Our approach could be a useful predictive/diagnostic tool; i.e., one could test whether WGS data from a new subject could contribute to determine whether that subject would be within or close to an existing MDD or control cluster. Advances in this line of research have the potential to be rapidly translated to clinical practice and could include the ability to diagnose patients based on WGS data.

## Additional Information

**How to cite this article:** Yu, C. *et al*. A novel strategy for clustering major depression individuals using whole-genome sequencing variant data. *Sci. Rep.* **7**, 44389; doi: 10.1038/srep44389 (2017).

**Publisher's note:** Springer Nature remains neutral with regard to jurisdictional claims in published maps and institutional affiliations.

## Supplementary Material

Supplementary Information

## Figures and Tables

**Table 1:** Whole-genome sequencing variation analysis of 25 human subjects.

Subjects	Gender	Age	Total SNVs	Total INDELs	dbSNP
MA-Depresson-1	Female	35	8,348,095	522,994	4,031,869
MA-Depresson-2	Female	30	7,921,961	513,462	3,993,392
MA-Depresson-3	Female	41	8,037,674	514,135	3,986,882
MA-Depresson-4	Female	32	8,021,058	511,756	3,903,495
MA-Depresson-5	Female	45	7,839,942	511,053	4,001,897
MA-Depresson-6	Female	38	7,834,986	516,002	4,021,724
MA-Depresson-7	Female	36	7,935,708	512,681	3,911,549
MA-Depresson-8	Female	59	7,694,178	514,095	3,949,370
MA-Depresson-9	Female	41	7,778,564	520,337	3,987,191
MA-Depresson-10	Female	31	8,073,958	526,792	4,045,542
MA-Control-1	Female	50	7,879,192	519,009	4,042,162
MA-Control-2	Female	45	6,974,138	517,756	4,021,858
MA-Control-3	Female	39	6,911,665	526,897	3,999,059
MA-Control-4	Female	29	7,197,066	518,675	4,011,644
MA-Control-5	Female	35	7,487,135	517,667	4,031,216
AU-Depresson-1	Male	44	3,883,255	555,785	3,888,831
AU-Depresson-2	Female	19	3,938,868	541,109	3,956,682
AU-Depresson-3	Female	19	3,925,906	560,127	3,928,997
AU-Depresson-4	Female	25	3,933,654	557,712	3,935,804
AU-Depresson-5	Female	18	3,905,386	555,542	3,920,378
AU-Control-1	Female	20	3,898,847	569,129	3,923,876
AU-Control-2	Male	18	3,920,681	558,496	3,903,217
AU-Control-3	Male	30	3,861,132	552,110	3,861,584
AU-Control-4	Female	18	3,922,531	568,501	3,911,346
AU-Control-5	Male	20	3,820,520	449,055	3,773,974

MA, Mexican-American; AU, Australian; SNVs, single nucleotide variants; INDELs, small insertions and deletions; dbSNP (the number of SNVs and INDELs that are found in the dbSNP database in NCBI).
