# Supplementary material for: A novel strategy for clustering major depression individuals using whole-genome sequencing variant data
Source: Sci Rep. 2017 Mar 13;7:44389. doi: 10.1038/srep44389 (PMC5347377; doi:10.1038/srep44389)
Supplement: Supplementary Information [file srep44389-s1.pdf]

A novel strategy for clustering major depression individuals using whole-genome sequencing variant data

Supplementary Information

Chenglong Yu, Bernhard T. Baune, Julio Licinio & Ma-Li Wong

Whole-Genome Sequencing (WGS) and Analysis

Fifteen Mexican-American subjects were whole-genome sequenced using Illumina HiSeq 2000 (BGI-Shenzhen, Shenzhen, Guangdong, China) and ten European-Ancestry Australian subjects were whole-genome sequenced using HiSeq X (Garvan Institute, Sydney, New South Wales, Australia). The read length for Mexican-American subjects is 151 and the read length for Australian subjects is 90. All the sequencing reads passed the quality control by evaluation of FastQC (www.bioinformatics.babraham.ac.uk/projects/fastqc/).

Firstly, we aligned the paired-end reads of each subject to the human reference genome (hg19, Genome Reference Consortium GRCh37) using Burrows-Wheeler Aligner (BWA) to obtain SAM format files, which were then converted to BAM format files using SAMtools. For example, align one sample of WGS paired-end reads (e.g., READS_R1 and READS_R2) to hg19 using the following commands:

```
bwa index hg19.fa
bwa aln -t 24 hg19.fa READS_R1.fastq.gz -f READS_R1.sai
bwa aln -t 24 hg19.fa READS_R2.fastq.gz -f READS_R2.sai
bwa sampe -a 1000 -n 1 -N 0 READS_R1.sai READS_R2.sai READS_R1.fastq.gz
READS_R2.fastq.gz | samtools view -Sbh - > READS.bam
```

Secondly, BAM files were then sorted and merged into one BAM file using the

following commands. Note: Mexico-American subjects have 8 lanes of sequencing reads, while Australian subjects have 2 lanes of sequencing reads.

samtools sort READS.bam READS.sort

samtools merge READS.merge.bam READS.lane1.sort.bam READS.lane2.sort.bam … READS.lane8.sort.bam

samtools index READS.merge.bam

Until here, for one subject, no matter how many lanes of reads, it has two files associated with: READS.merge.bam and READS.merge.bam.bai.

Thirdly, mpileup command in SAMtools was applied to calculate the likelihood of data given each possible genotype, and stored the likelihoods in a binary file. The output was supplied to SAMtools/BCFtools which created SNV and INDEL calling to produce VCF files, as follows:

samtools mpileup -m 3 -F 0.0002 -E -uf READS.merge.bam | bcftools view -bvcg - > READS.merge.raw.bcf

(-m: minimum number gapped reads for indel candidates; -F: minimum fraction of gapped reads)

bcftools view READS.merge.raw.bcf | vcfutils.pl varFilter -D10000 > READS.merge.flt.vcf

(-D: the maximum read depth to call a SNP)

Finally, ANNOVAR software was used to annotate SNV/INDEL information and classifications such as their located chromosome, position, genetic region, etc.

The same filtering criteria and quality thresholds used above are performed in this study. All WGS analyses were performed using high performance computers in eResearch South Australia (www.ersa.edu.au).

Table S1: The SNV distributions for all the chromosomes and mitochondrial genome on 25 subjects.

Subjects	chr1	chr2	chr3	chr4	chr5	chr6	chr7
AU-Depression-1	301103	309712	257417	280613	232115	224475	220584
AU-Depression-2	302345	308879	258428	281812	230408	227551	223214
AU-Depression-3	300643	313677	257598	277705	228690	228731	217858
AU-Depression-4	295673	308585	257864	285079	231252	225732	218744
AU-Depression-5	295499	309589	259592	278681	231401	225789	217576
AU-Control-1	297701	311754	252715	278477	228314	223086	219690
AU-Control-2	298129	316209	264304	280435	234834	226494	226061
AU-Control-3	297444	310873	253499	276636	230161	223149	221053
AU-Control-4	301728	312226	258565	281661	222430	231444	219742
AU-Control-5	299695	303323	252077	279923	227056	226770	214973
MA-Depression-1	679766	645735	524762	495349	461734	448583	462339
MA-Depression-2	639621	616461	495345	481095	437194	419596	439435
MA-Depression-3	658412	622542	497347	482736	443255	425832	446506
MA-Depression-4	655935	619597	499050	475129	437081	439003	444769
MA-Depression-5	632081	608478	488763	469599	431336	417863	435013
MA-Depression-6	634558	608724	491261	470333	429661	426306	437679
MA-Depression-7	649148	617308	500847	472364	441907	429721	438998
MA-Depression-8	621592	585536	484652	463909	420754	417845	427428
MA-Depression-9	635500	606553	494412	473792	430628	417690	435011
MA-Depression-10	658107	619897	508103	477713	449647	453626	450334
MA-Control-1	630447	602339	487642	487690	444983	426340	441277
MA-Control-2	557708	543372	441458	432110	392866	382895	390751
MA-Control-3	554411	535557	429800	424226	382304	376376	379690
MA-Control-4	574542	556280	458368	442338	400717	390948	403074
MA-Control-5	600317	582748	477077	457213	419489	406146	409664

Cont'd

Subjects	chr8	chr9	chr10	chr11	chr12	chr13	chr14	chr15
AU-Depression-1	199951	166209	189236	194160	176898	142749	124289	116527
AU-Depression-2	203690	168932	194438	190806	177700	145497	120838	115771
AU-Depression-3	204146	167988	194672	198832	176875	142126	122030	112719
AU-Depression-4	200218	168508	193294	195582	180722	144535	122855	110824
AU-Depression-5	202668	167979	184994	190222	176929	142061	127249	113120
AU-Control-1	198762	163138	194487	197711	175339	143577	123786	112916
AU-Control-2	204044	170967	187319	194767	180736	143947	121725	112966
AU-Control-3	201758	168012	190355	193074	176021	142611	121409	111465
AU-Control-4	198833	167497	191316	185471	176975	143964	122564	116204
AU-Control-5	192594	164207	189857	187084	175067	141986	118303	110360
MA-Depression-1	388959	346014	403710	403719	383599	256596	261939	249474
MA-Depression-2	378224	334776	383721	382226	371303	247321	243704	245237
MA-Depression-3	382347	345383	391092	391628	370823	250641	252677	238754
MA-Depression-4	378379	334342	382310	387161	374687	252134	251719	246359
MA-Depression-5	372578	327002	378750	379221	365658	249826	248145	238535
MA-Depression-6	374907	331984	375119	383156	365836	243267	244917	235552
MA-Depression-7	378682	328864	380992	384237	370497	252298	255155	233729
MA-Depression-8	372118	322243	367333	371837	361448	242113	242382	225683
MA-Depression-9	357904	322703	373350	368774	364080	243454	253001	233471
MA-Depression-10	378306	335510	391803	385678	382182	259090	251310	235031
MA-Control-1	371106	327109	375211	386518	367995	246994	248941	232308
MA-Control-2	336789	297635	332121	336995	322986	229837	218757	205444
MA-Control-3	335511	291832	333755	342052	320353	221824	218206	202795
MA-Control-4	345336	305764	346363	348015	330254	234331	226578	213957
MA-Control-5	372216	318105	354298	357862	344854	247261	238118	219509

Cont'd

Subjects	chr16	chr17	chr18	chr19	chr20	chr21	chr22	chrX	mitoch.
AU-Depression-1	123279	103626	104959	86369	83018	61877	53849	85949	40
AU-Depression-2	124506	105457	111056	85690	87241	61711	52871	111473	33
AU-Depression-3	118640	103020	108004	88157	85516	63612	52333	113406	42
AU-Depression-4	124443	105421	107476	88312	86334	65999	53507	110517	45
AU-Depression-5	120287	104550	109791	85490	84111	63396	53973	110009	37
AU-Control-1	122668	101500	106196	89819	83122	64048	52846	108968	31
AU-Control-2	123882	106762	105875	86376	84885	62649	53529	88594	41
AU-Control-3	118510	103761	106808	86074	85458	61578	52225	86203	36
AU-Control-4	122476	105047	109143	83104	86669	63905	55143	110453	45
AU-Control-5	117170	101448	109008	84786	83752	62618	52848	83601	34
MA-Depression-1	296951	310799	209773	285379	208912	115886	157214	299345	40
MA-Depression-2	279068	288149	200637	265679	195608	113335	142242	270017	45
MA-Depression-3	284439	289549	199756	270495	198247	113783	144935	283100	35
MA-Depression-4	278849	298315	200454	268647	197554	113432	145509	285835	38
MA-Depression-5	279323	281794	200208	265182	193834	110750	142277	270513	35
MA-Depression-6	276348	283022	193048	258249	190593	110934	139562	274107	39
MA-Depression-7	274952	284617	196695	266285	195801	111948	143798	273209	38
MA-Depression-8	270432	280267	196849	259618	185984	114817	137540	269478	32
MA-Depression-9	272615	278462	195224	259835	194107	110746	140040	262722	36
MA-Depression-10	276956	287267	203351	262356	202473	115314	146195	288671	33
MA-Control-1	272094	284954	195064	266710	195962	112194	140603	282901	33
MA-Control-2	239736	238248	176990	220995	168016	102510	119637	231986	33
MA-Control-3	244220	241203	175546	223926	170704	104209	121759	226966	41
MA-Control-4	250721	250301	179152	233122	174939	104086	126194	245614	31
MA-Control-5	258634	270134	188544	237801	179594	107697	129601	254991	47

Figure S1: The phylogenetic clustering trees for 25 subjects on chromosomes 2, 3, 4, 5, 6, 7, 8, 9, 10, 11, 12, 13, 14, 15, 16, 17, 18, 19, 20, 21, and mitochondrion. Except the mitochondrial genome, all the other trees can well cluster Mexican-American MDD patients together.

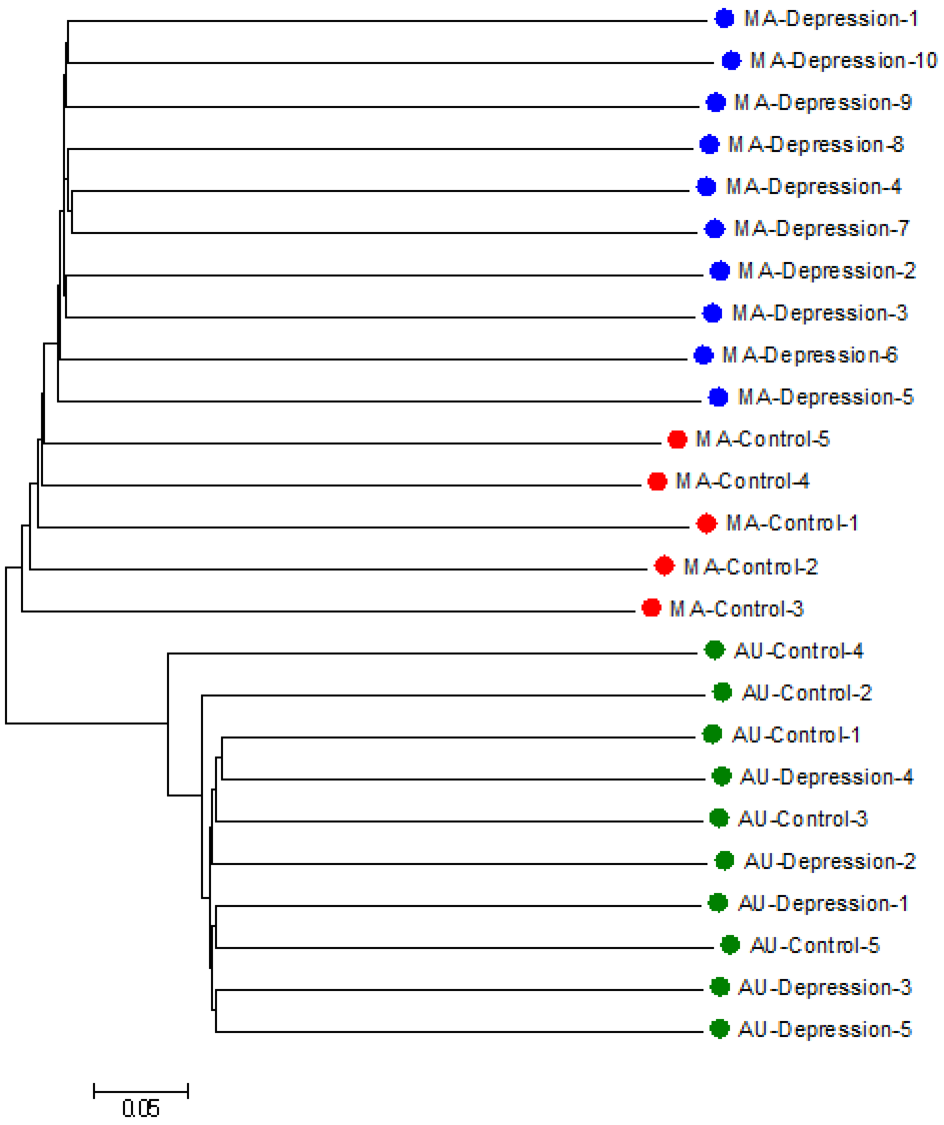

Chromosome 2

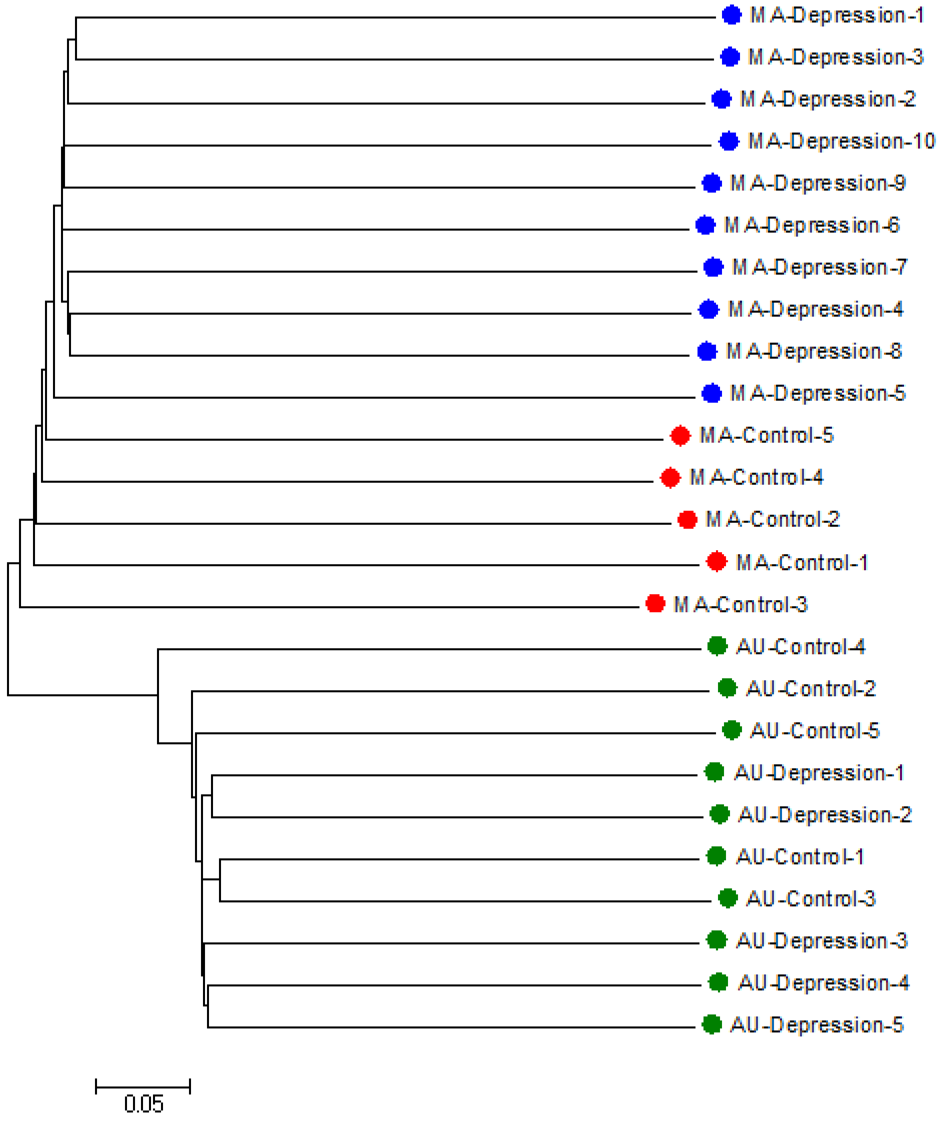

Chromosome 3

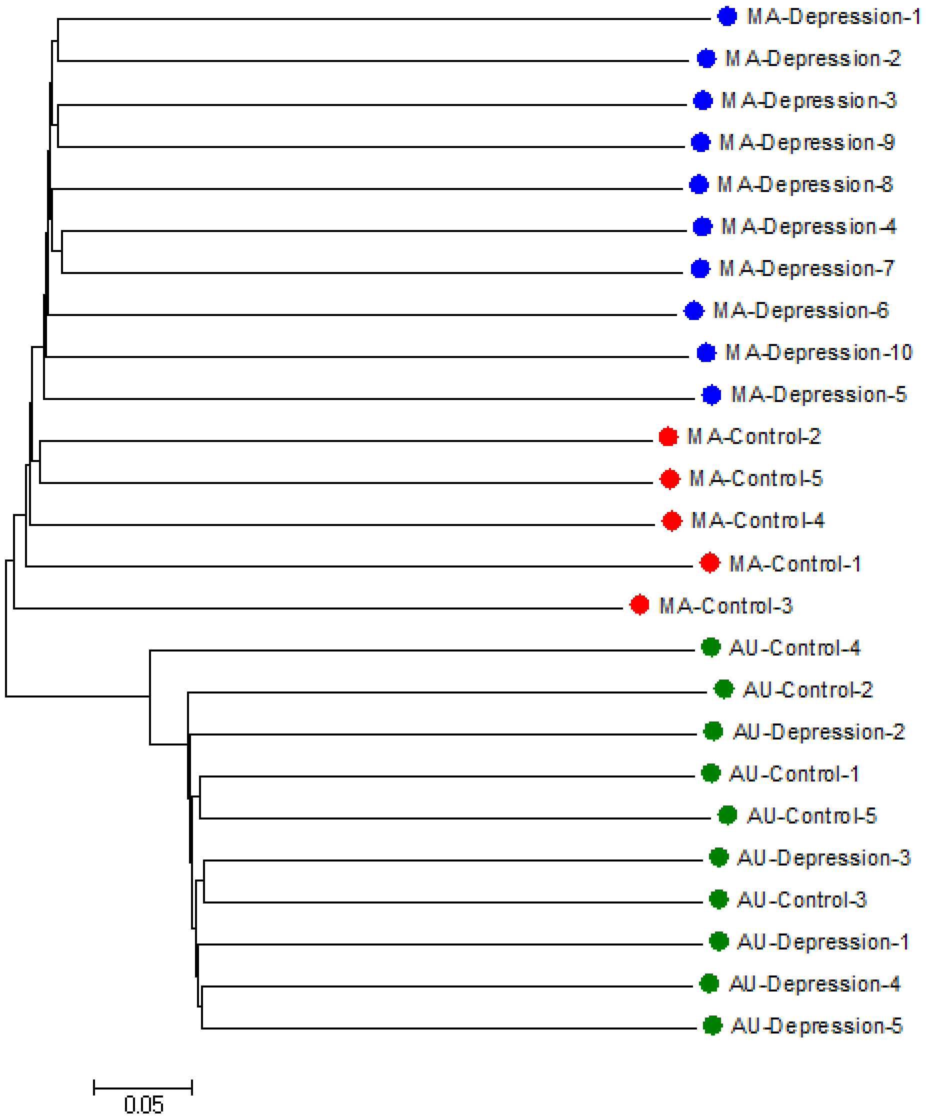

Chromosome 4

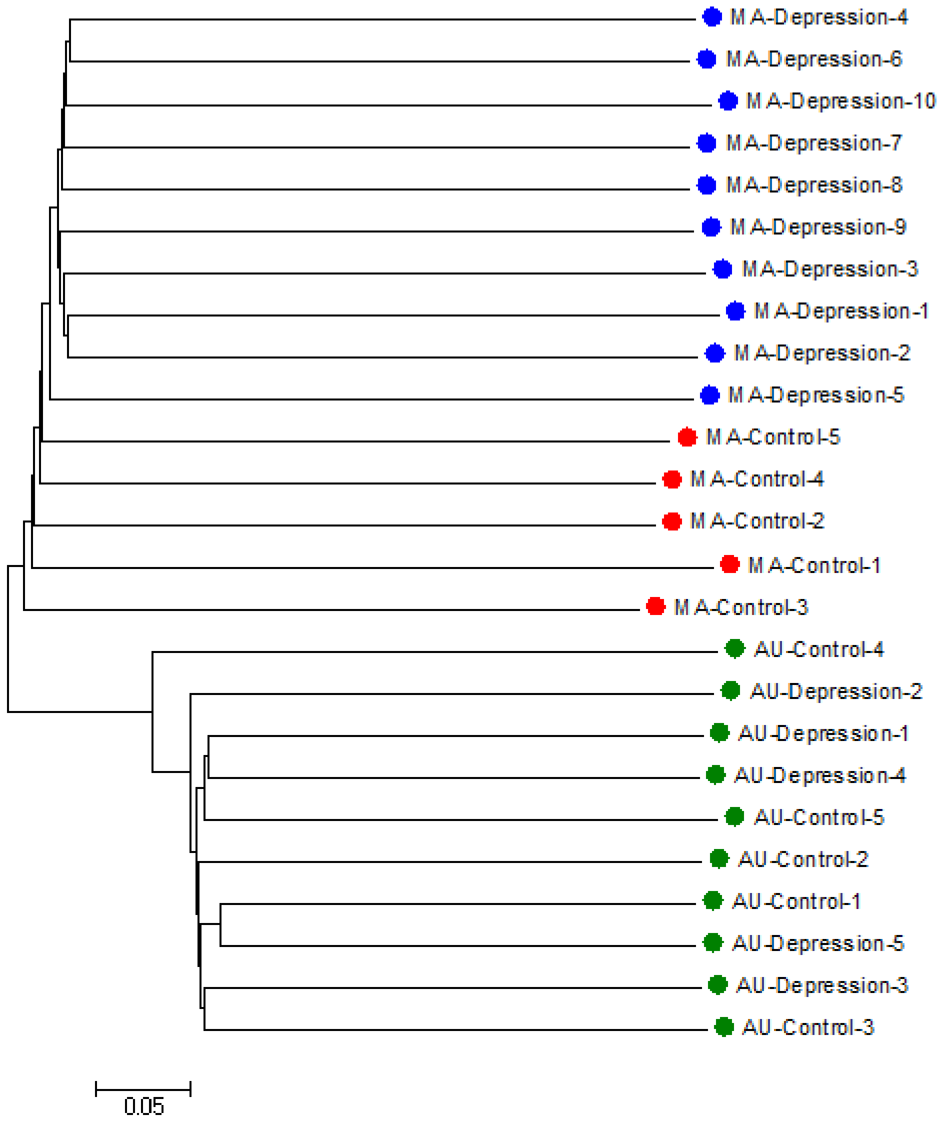

Chromosome 5

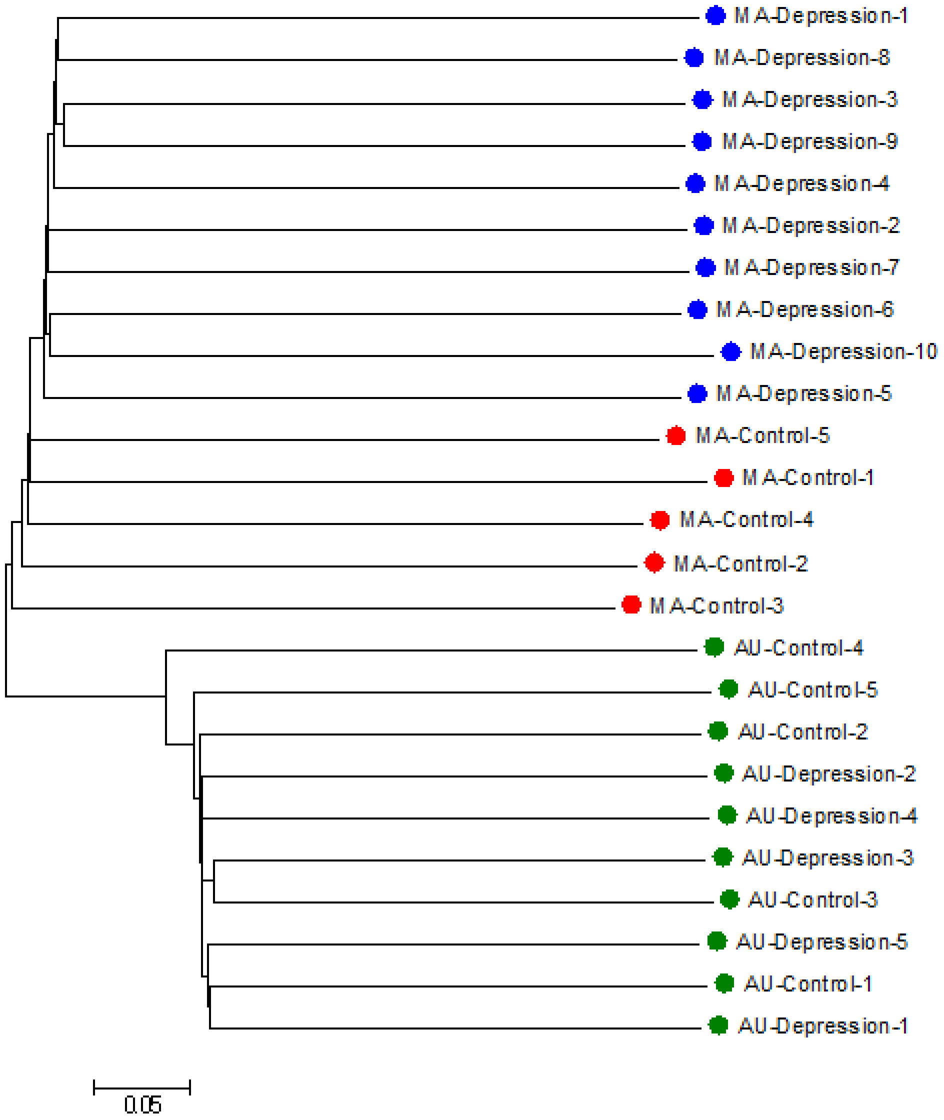

Chromosome 6

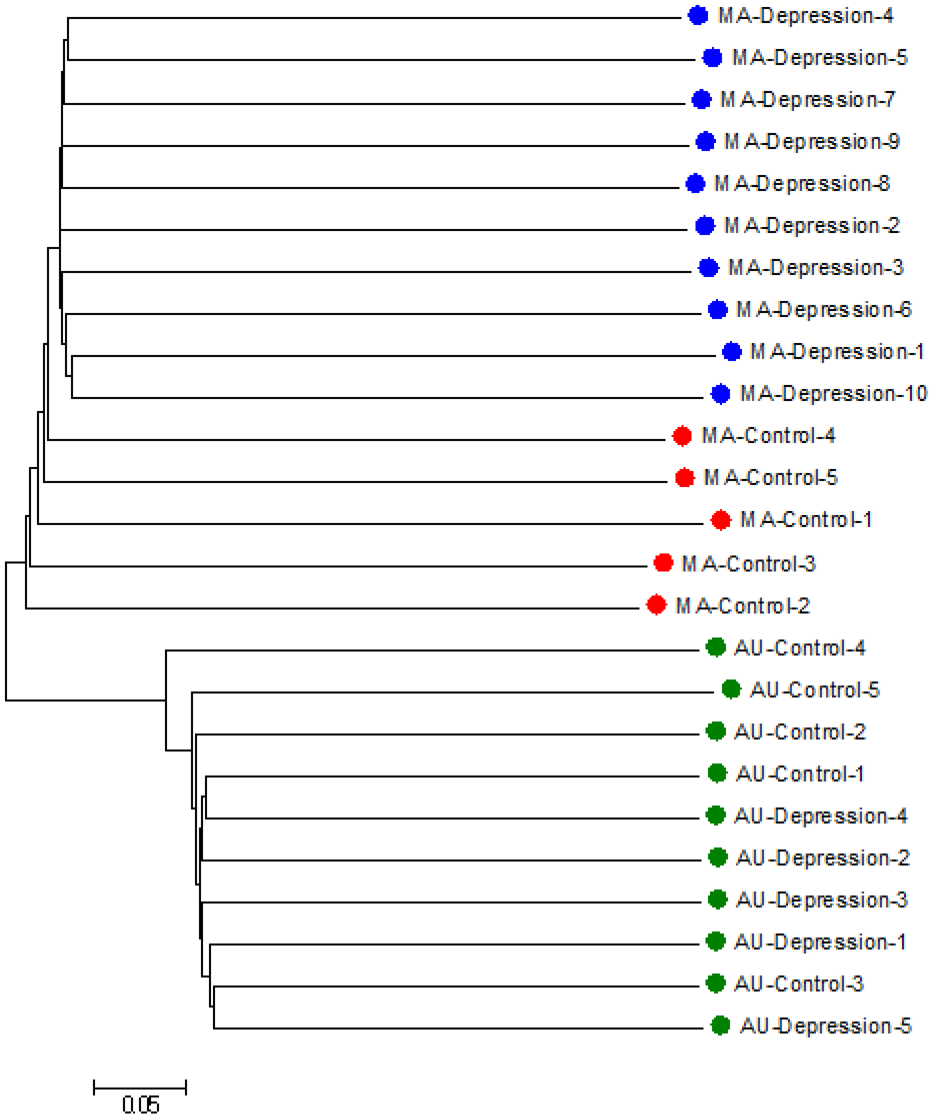

Chromosome 7

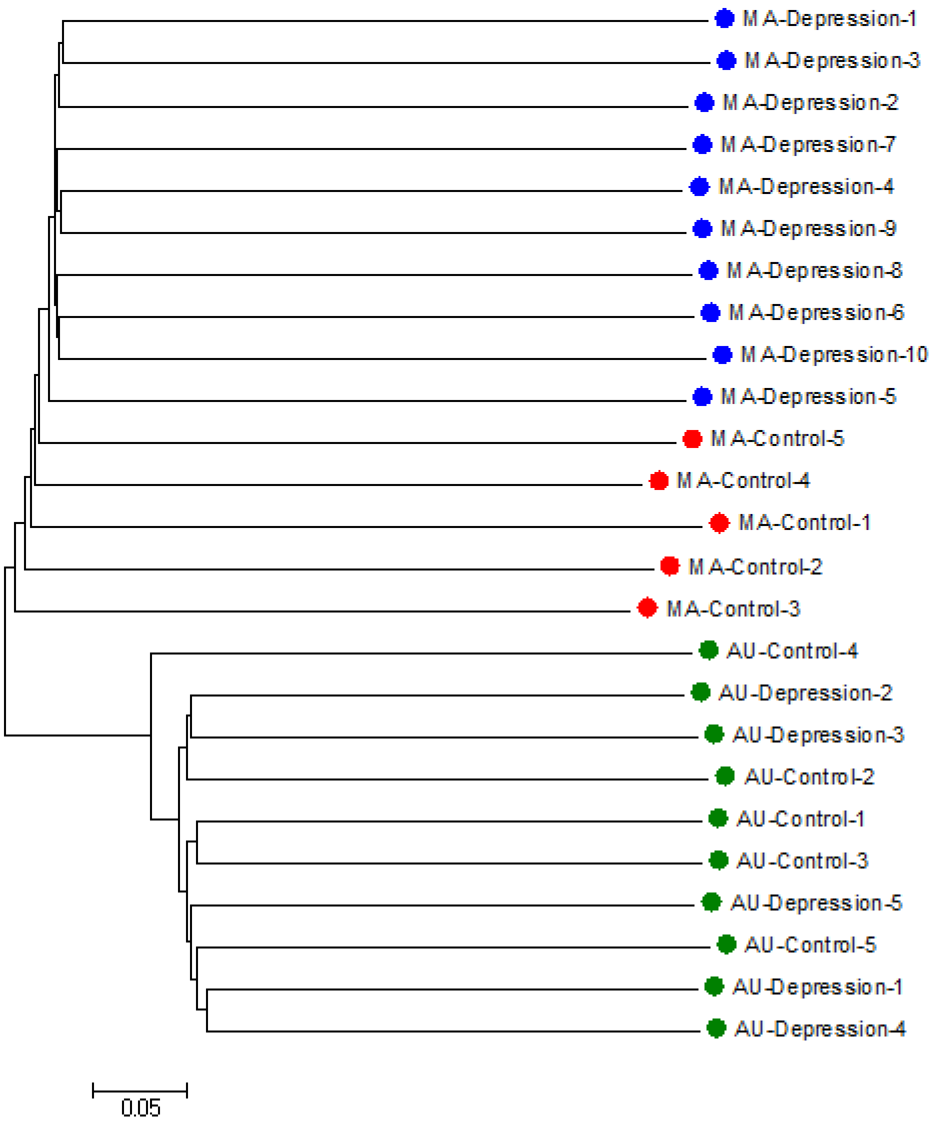

Chromosome 8

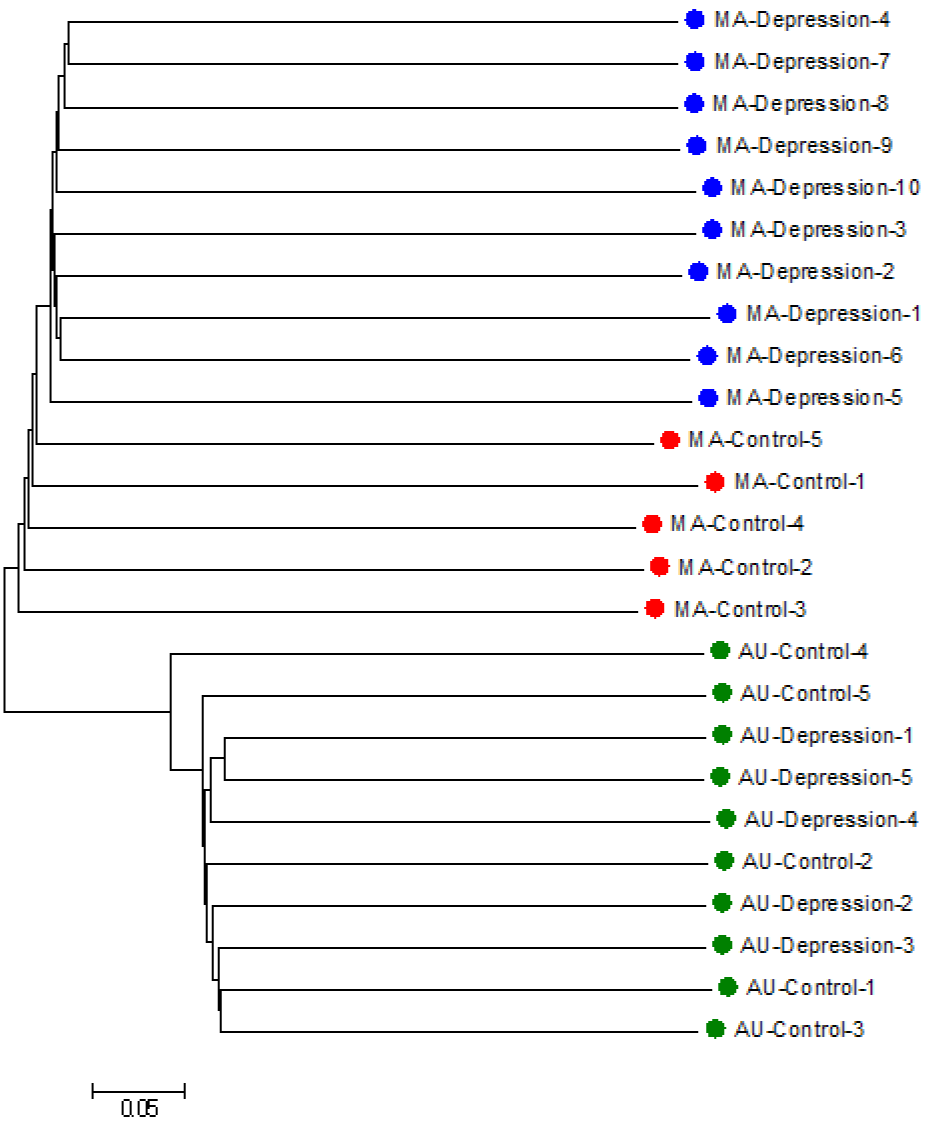

Chromosome 9

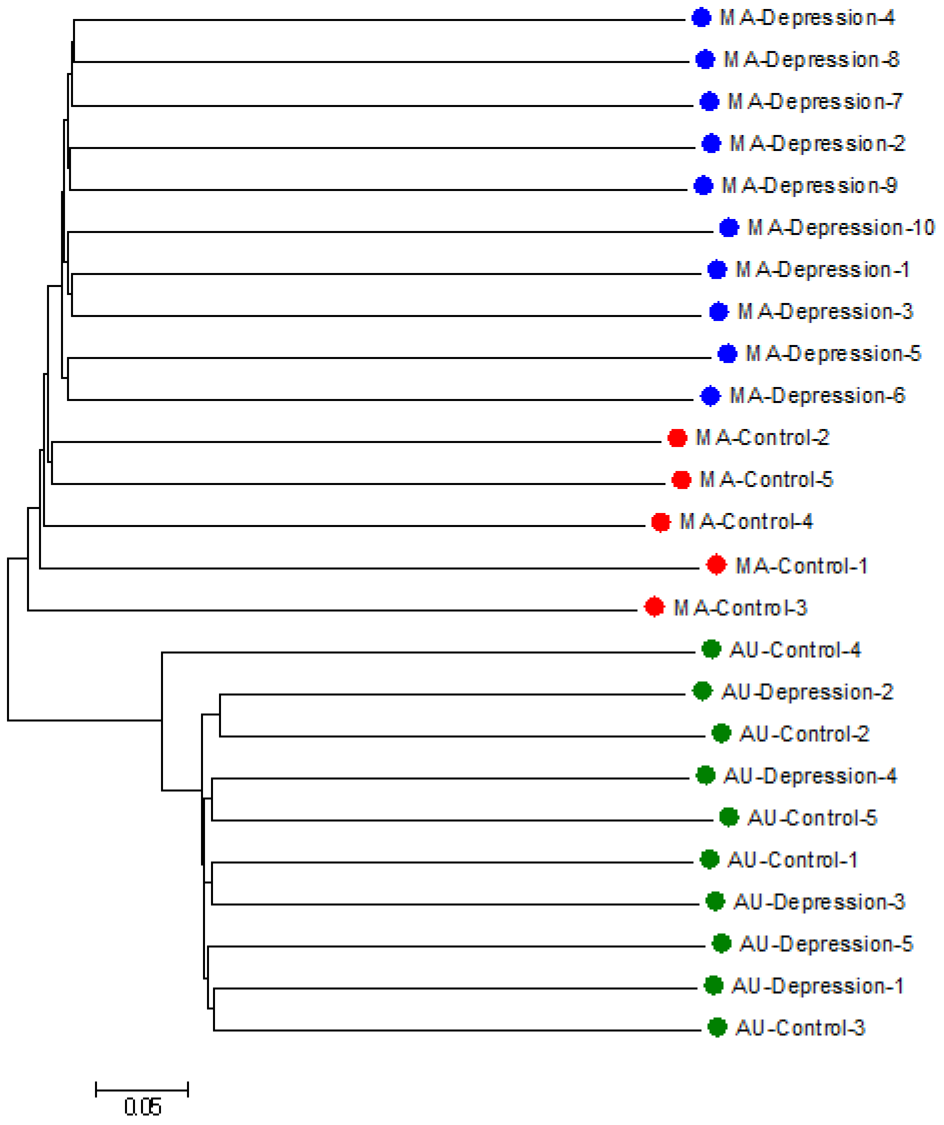

Chromosome 10

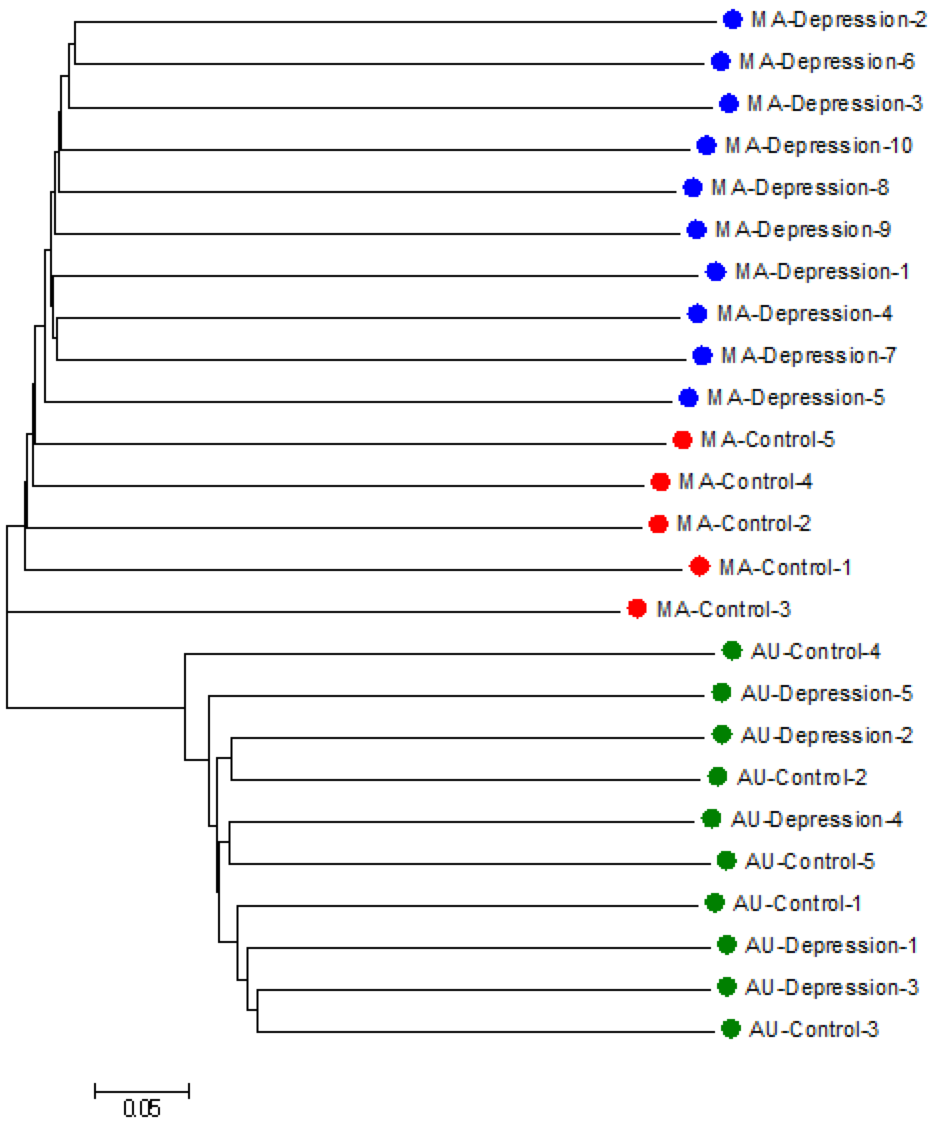

Chromosome 11

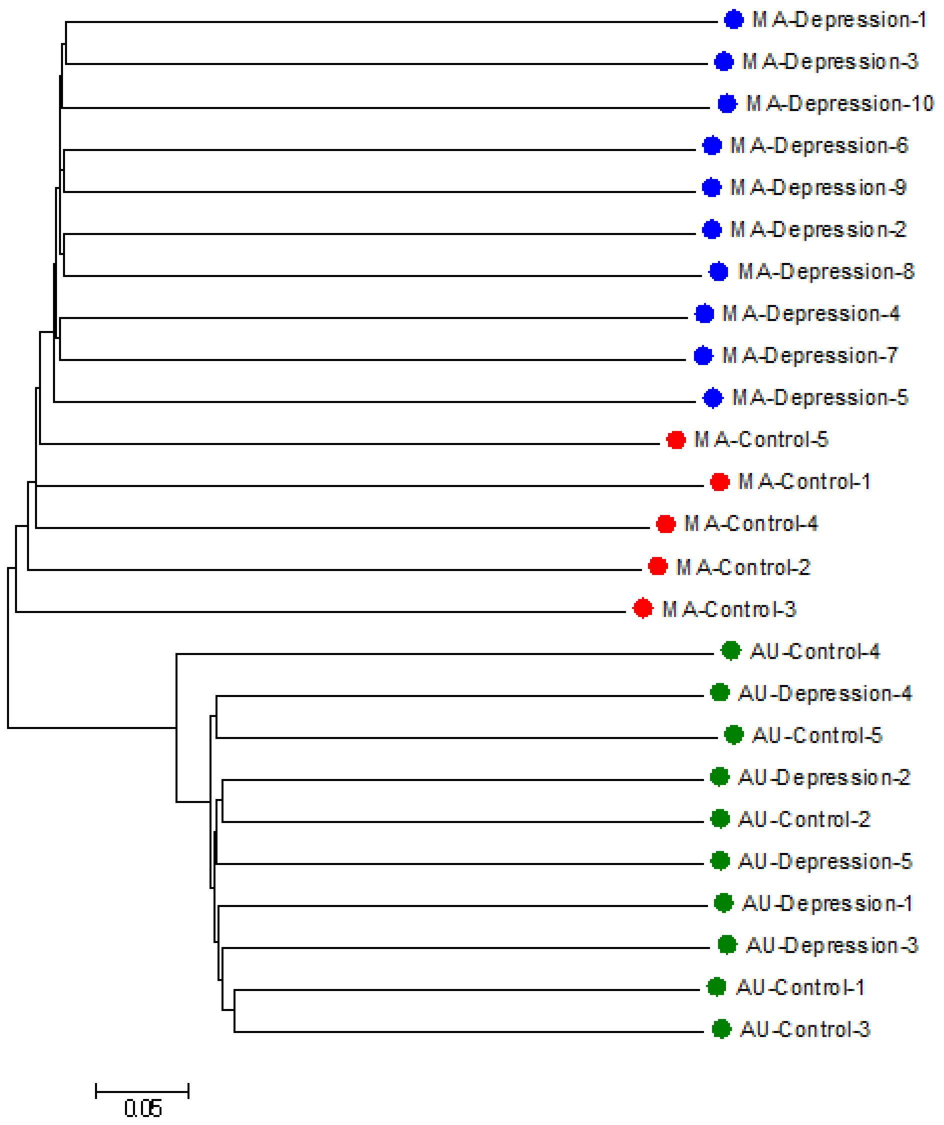

Chromosome 12

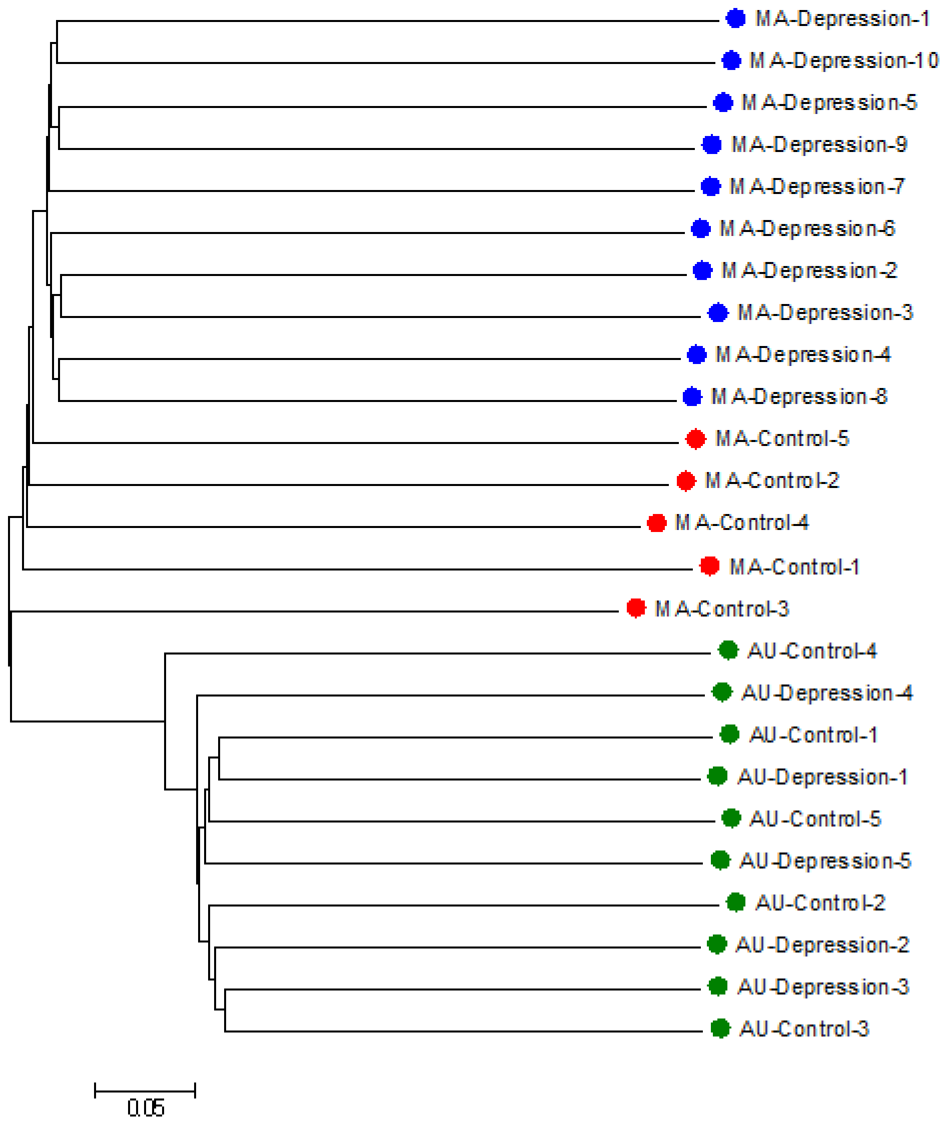

Chromosome 13

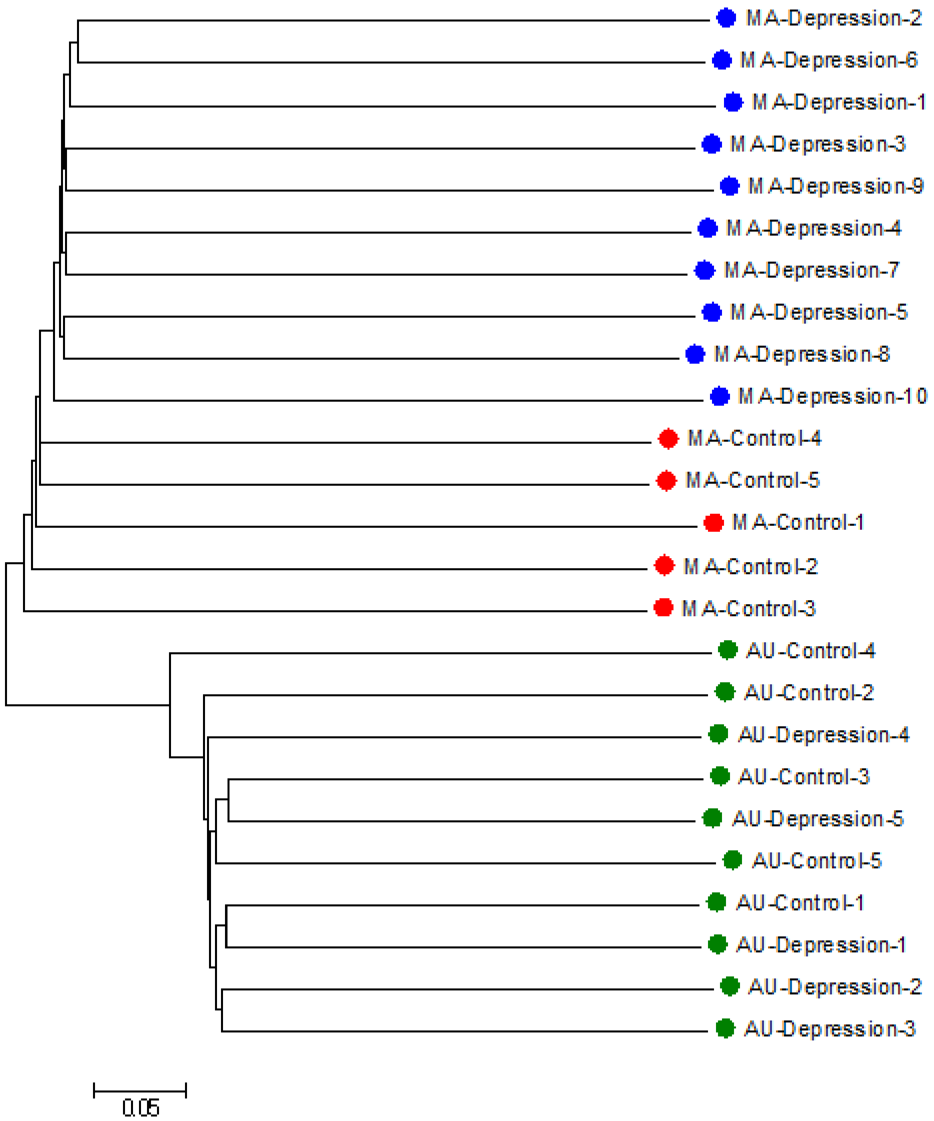

Chromosome 14

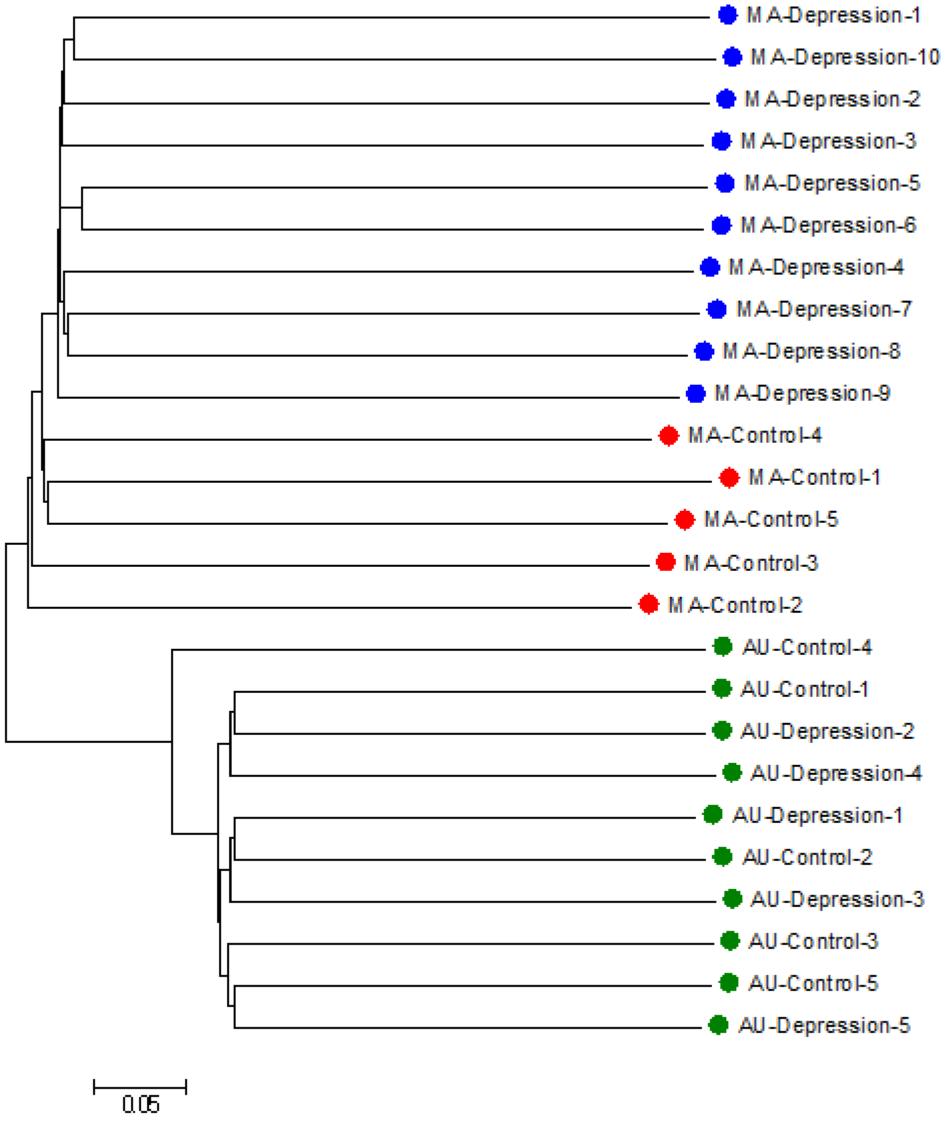

Chromosome 15

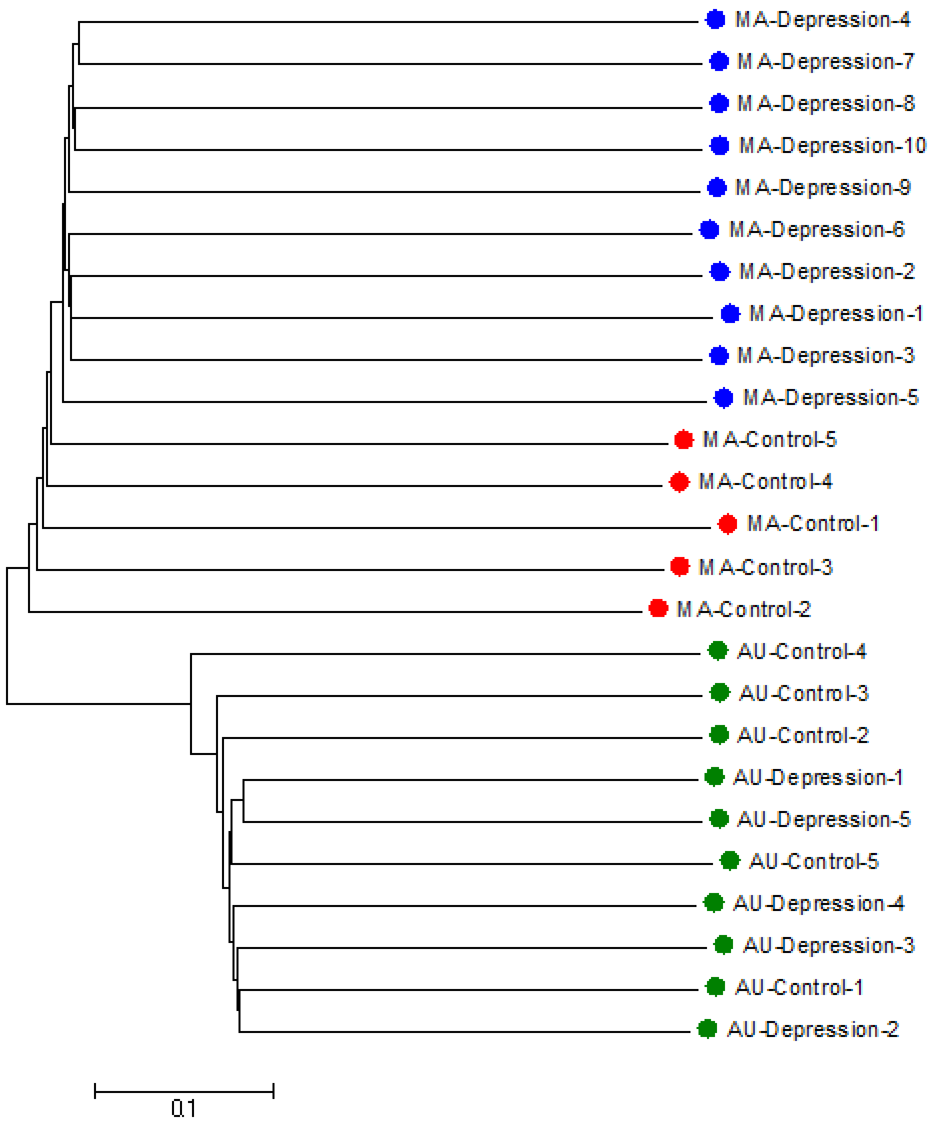

Chromosome 16

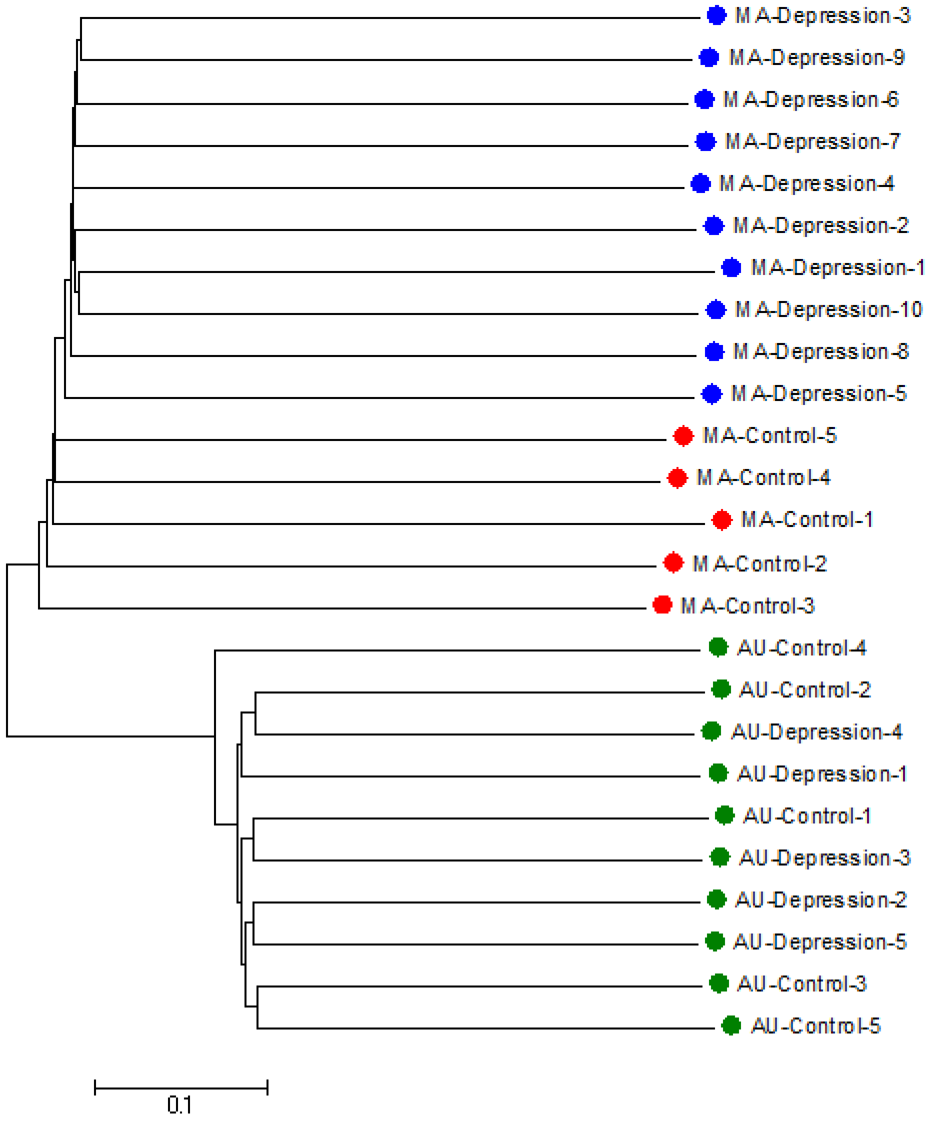

Chromosome 17

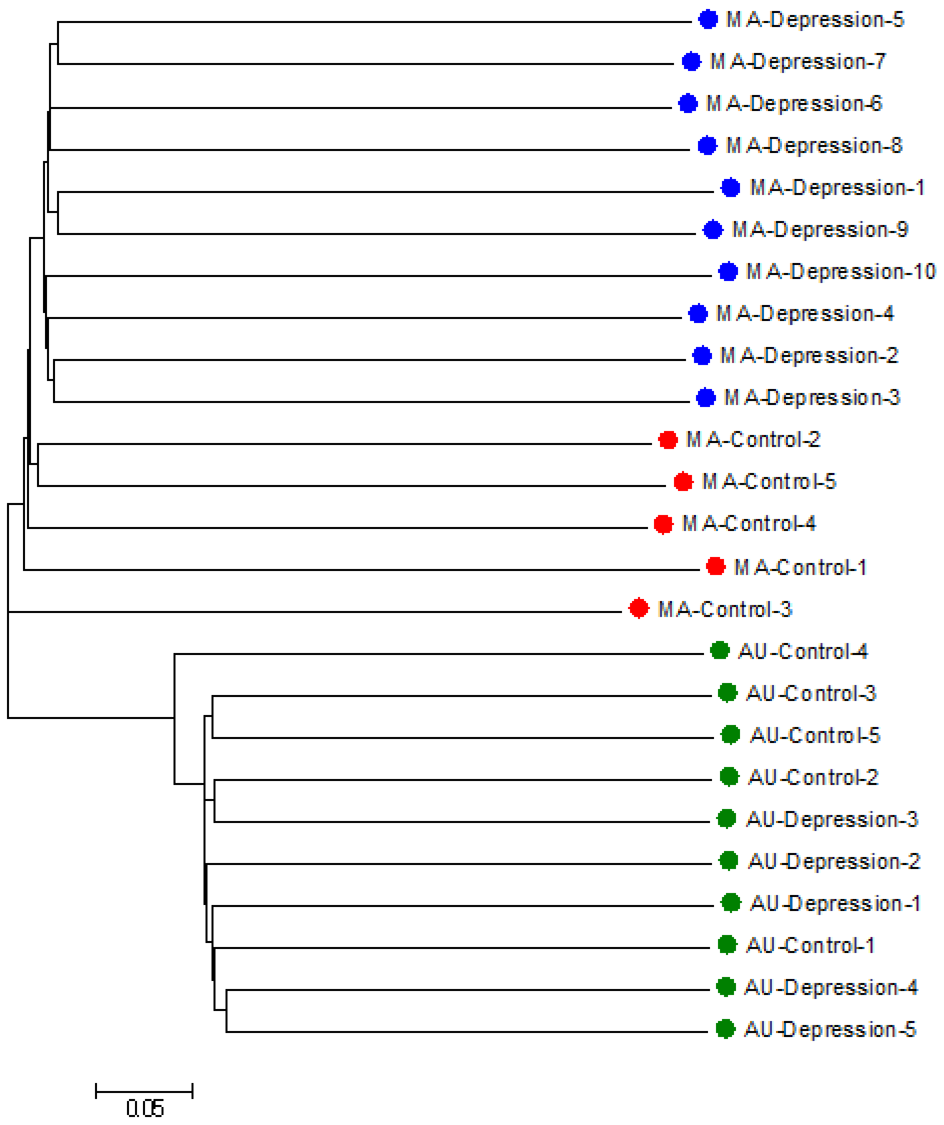

Chromosome 18

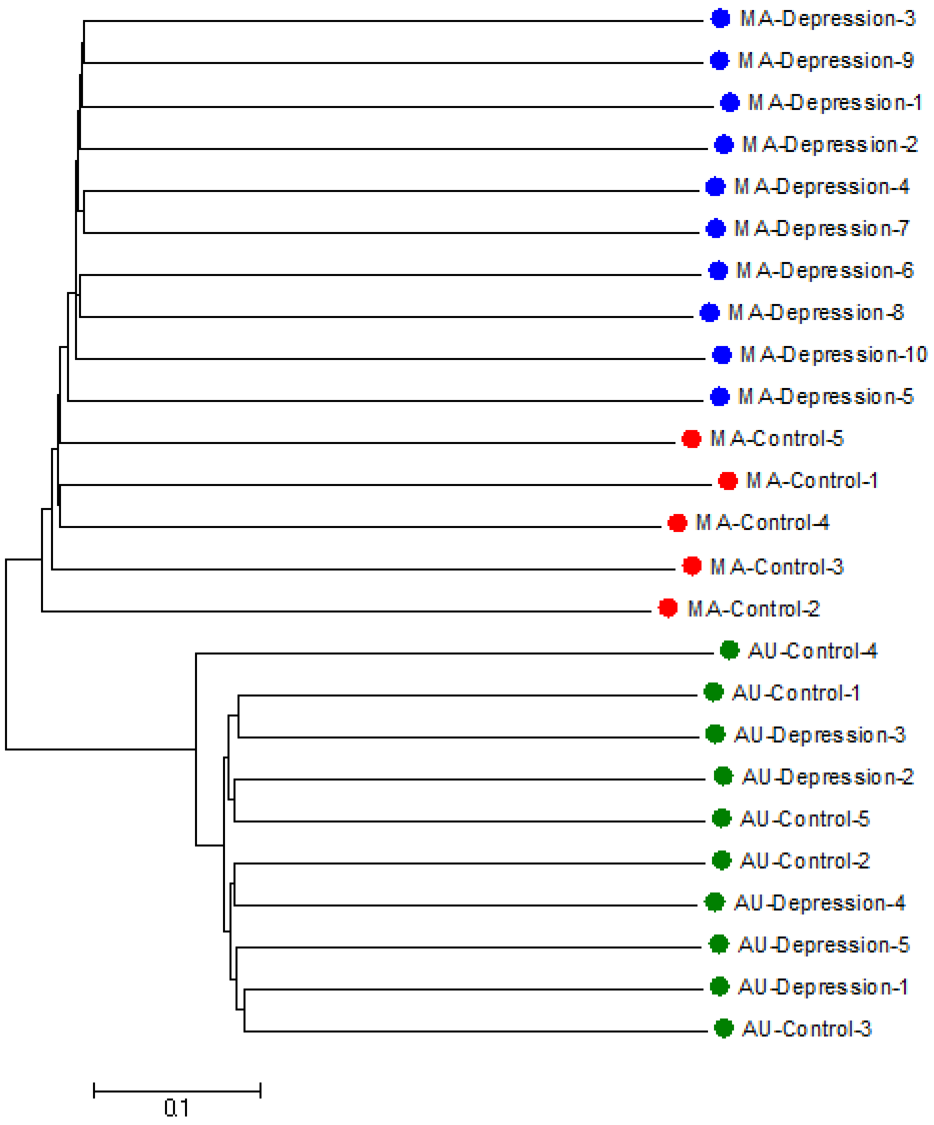

Chromosome 19

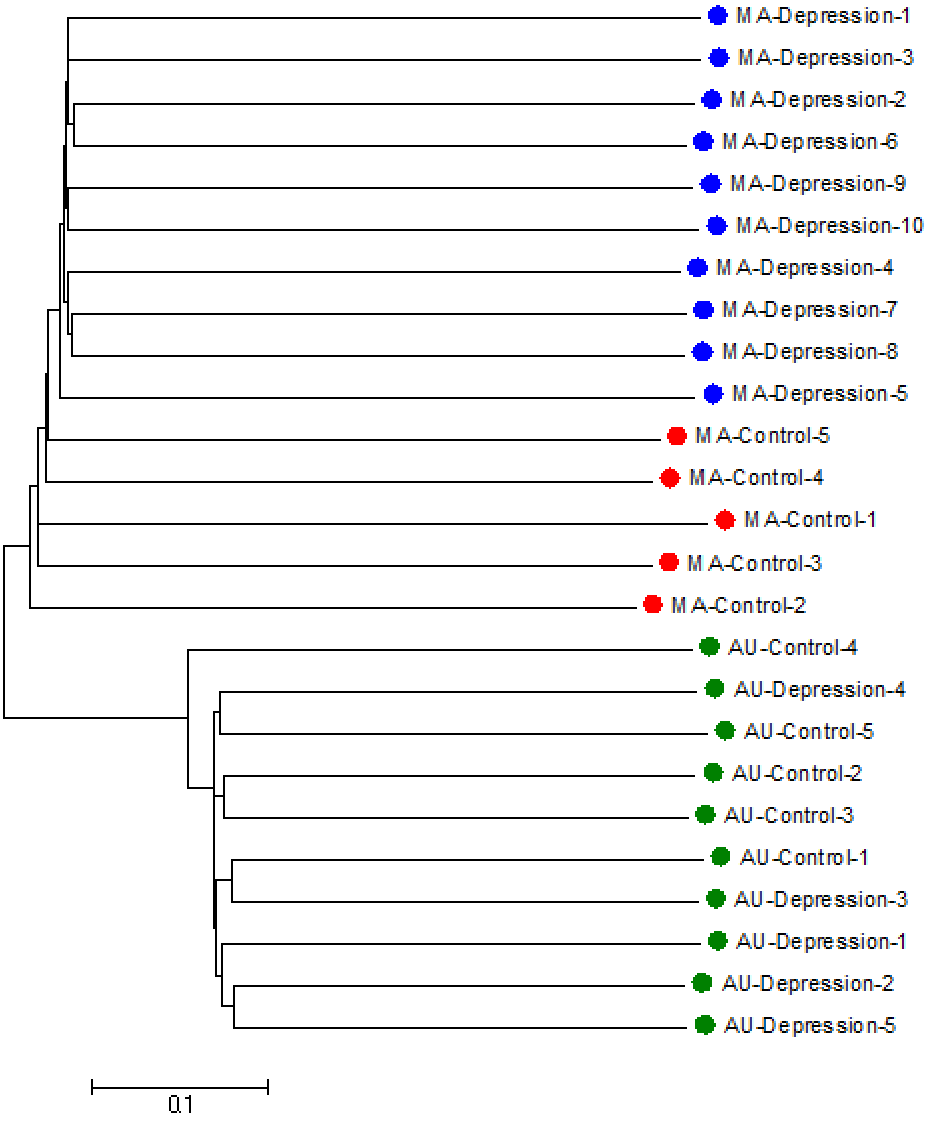

Chromosome 20

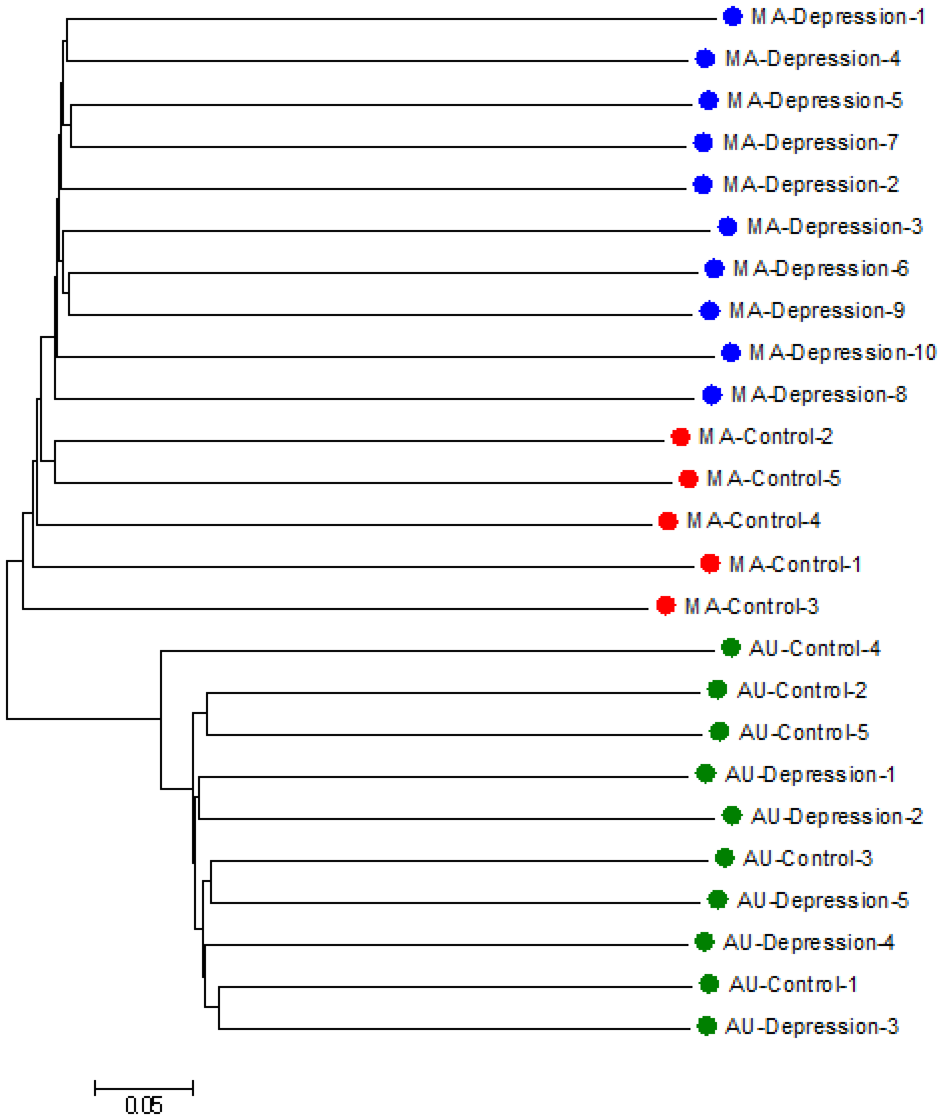

Chromosome 21

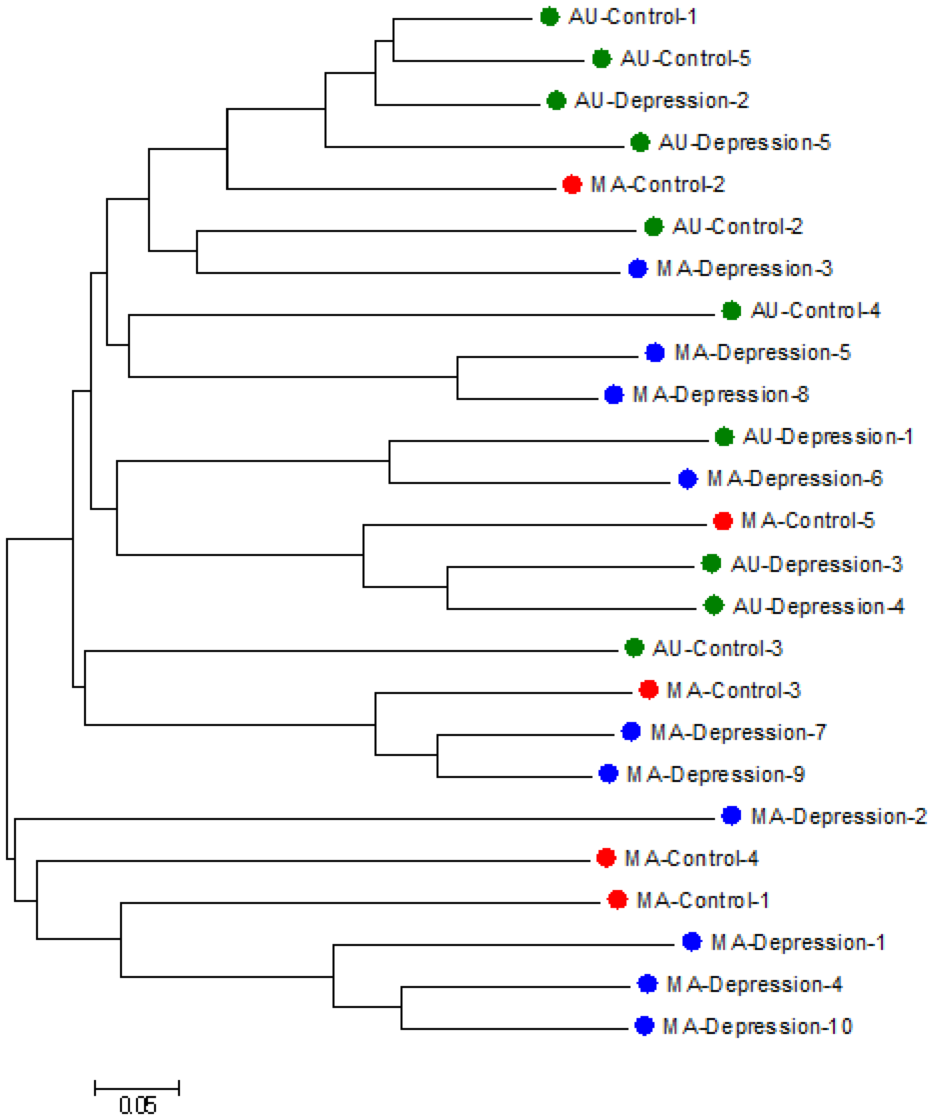

Mitochondrial genome
